# Isolation, production and characterization of fully human monoclonal antibodies directed to *Plasmodium falciparum* MSP10

**DOI:** 10.1186/s12936-015-0797-x

**Published:** 2015-07-16

**Authors:** Dominika J Maskus, Susanne Bethke, Melanie Seidel, Stephanie Kapelski, Otchere Addai-Mensah, Alexander Boes, Güven Edgü, Holger Spiegel, Andreas Reimann, Rainer Fischer, Stefan Barth, Torsten Klockenbring, Rolf Fendel

**Affiliations:** Fraunhofer Institute for Molecular Biology and Applied Ecology IME, Aachen, Germany; Institute for Molecular Biotechnology, RWTH Aachen University, Aachen, Germany; Faculty of Allied Health Sciences, Kwame Nkrumah University of Science and Technology, Kumasi, Ghana; Department of Experimental Medicine and Immunotherapy, Institute for Applied Medical Engineering at RWTH Aachen University and Hospital, Aachen, Germany

**Keywords:** EBV transformation, Human monoclonal antibodies, *Plasmodium falciparum* MSP10, Plant-based expression

## Abstract

**Background:**

Semi-immunity against the malaria parasite is defined by a protection against clinical episodes of malaria and is partially mediated by a repertoire of inhibitory antibodies directed against the blood stage of *Plasmodium falciparum*, in particular against surface proteins of merozoites, the invasive form of the parasite. Such antibodies may be used for preventive or therapeutic treatment of *P.falciparum* malaria. Here, the isolation and characterization of novel human monoclonal antibodies (humAbs) for such applications is described.

**Methods:**

B lymphocytes had been selected by flow cytometry for specificity against merozoite surface proteins, including the merozoite surface protein 10 (MSP10). After Epstein-Barr virus (EBV) transformation and identification of promising resulting lymphoblastoid cell lines (LCLs), human immunoglobulin heavy and light chain variable regions (Vh or Vl regions) were secured, cloned into plant expression vectors and transiently produced in *Nicotiana benthamiana* in the context of human full-size IgG1:κ. The specificity and the affinity of the generated antibodies were assessed by ELISA, dotblot and surface plasmon resonance (SPR) spectroscopy. The growth inhibitory activity was evaluated based on growth inhibition assays (GIAs) using the parasite strain 3D7A.

**Results:**

Supernatants from two LCLs, 5E8 and 5F6, showed reactivity against the second (5E8) or first (5F6) epidermal growth factor (EGF)-like domain of MSP10. The isolated V regions were recombinantly expressed in their natural pairing as well as in combination with each other. The resulting recombinant humAbs showed affinities of 9.27 × 10^−7^ M [humAb10.1 (H5F6:κ5E8)], 5.46 × 10^−9^ M [humAb10.2 (H5F6:κ5F6)] and 4.34 × 10^−9^ M [humAb10.3 (H5E8:κ5E8)]. In GIAs, these antibodies exhibited EC_50_ values of 4.1 mg/ml [95% confidence interval (CI) 2.6–6.6 mg/ml], 6.9 mg/ml (CI 5.5–8.6 mg/ml) and 9.5 mg/ml (CI 5.5–16.4 mg/ml), respectively.

**Conclusion:**

This report describes a platform for the isolation of human antibodies from semi-immune blood donors by EBV transformation and their subsequent characterization after transient expression in plants. To our knowledge, the presented antibodies are the first humAbs directed against *P. falciparum* MSP10 to be described. They recognize the EGF-like folds of MSP10 and bind these with high affinity. Moreover, these antibodies inhibit *P. falciparum* 3D7A growth in vitro.

## Background

Malaria still claims over half a million victims each year and poses a significant burden to global health care and to the economy of endemic countries [1]. In humans, semi-immunity to clinical malaria develops slowly due to high allelic variations in many immuno-relevant plasmodial antigens, is incomplete and wanes quickly without frequent reinfections [2–4]. Several lines of defense contribute to the immune response against the erythrocytic stages of plasmodia, e.g. innate-like Vγ9:Vδ2 T cells [5] and anti-plasmodial antibodies. The repertoire and spectrum of antibodies which eventually can prevent clinical episodes of malaria gradually develops with cumulative exposure to infections [6–8]. Passive transfer of naturally acquired antibodies has been shown to reduce parasitaemia in infected individuals [9, 10]. Such anti-plasmodial antibodies may mediate protection by prevention of re-invasion of merozoites into new erythrocytes [11], by antibody-dependent cellular inhibition mediated by monocytes [3, 12] and by antibody-dependent respiratory burst mediated by neutrophil granulocytes [13].

Most of the antibodies used for studies in the malaria field originate from rodents or rabbits. Unquestionably, these antibodies are valuable tools. However, they do not reflect the human immunoglobulin repertoire of semi-immune individuals. Malaria vaccine development is focusing on targets from the three major life cycle stages of the parasite in humans, the liver stage, the blood stage and the sexual stage. Sterile immunity, induced by experimental malaria infection, is mainly conferred by immune responses against the pre-erythrocytic stages [14]. However, immune responses in natural infection in hyper- and holoendemic regions are primarily focused on the blood stage and the protection is incomplete [15]. Targets of this immune response mediating partial protection are mainly surface proteins of the merozoite stage. Members of the merozoite surface protein (MSP) family, the reticulocyte homology (Rh) and the erythrocyte-binding like (EBL) proteins as well as the apical membrane antigen 1 (AMA1) play a central role. All of these proteins are in the focus of vaccine candidate studies [16]. One of the most recently identified members of the MSP family is MSP10. MSP10 was first described by Black et al., but until today there is little known about the function of this protein and whether it is essential [17]. Under the assumption that the genome of *Plasmodiumfalciparum* encodes more than one MSP that contains a double epidermal growth factor (EGF)-like domain, Black et al. sought to identify potential homologs of MSP1. It appears that MSP1 and MSP10 have several features in common; i.e. they share a double EGF-like domain and a glycosylphosphatidylinositol (GPI) anchor at their C-terminus and both are mainly expressed during the later blood stages. Furthermore, MSP10 is also subject to proteolytic processing similar to MSP1, i.e. in the case of MSP10 starting from 30 h post invasion a product of 36 kDa can be detected besides the intact protein of 80 kDa. Puentes et al. aimed at finding out if certain parts of MSP10 are capable of binding to erythrocytes and to inhibit the invasion of merozoites into new erythrocytes [18]. Three MSP10-derived 20-mer peptides showed these properties, thus arguing for a role of MSP10 in the attachment, re-orientation and/or invasion. Nevertheless, human monoclonal antibodies (humAbs) directed MSP10 have neither been generated nor characterized yet.

The aim of this work was to isolate MSP10-specific humAbs from semi-immune donors living in the Ashanti region in Ghana, a holoendemic area. Three humAbs, humAb10.1, humAb10.2 and humAb10.3, which are specific for either the first or the second EGF-like domain of MSP10, were isolated and characterized in detail.

## Methods

### Recombinant plasmodial proteins

**Figure 1:**
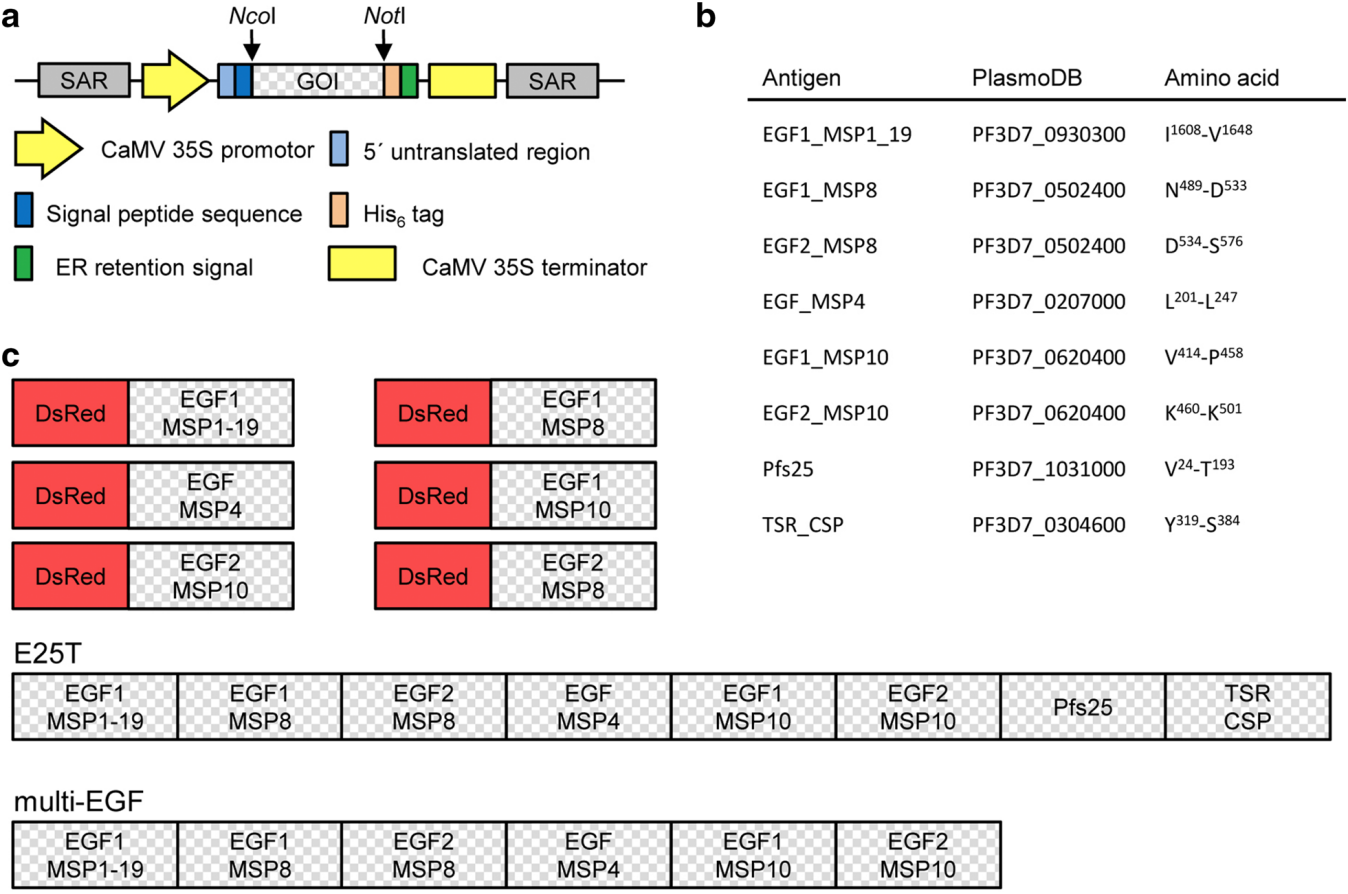
Schematic representation of the plant expression cassette and the construct design for the recombinant plasmodial antigens. The gene of interest (GOI) is expressed in *Nicotiana benthamiana* using the vector pTRAc with the shown expression cassette (**a**). The constructs used during this study are summarized including the PlasmoDB number and the amino acid sequences for the respective domains (**b**). Antigens were either expressed as single domain constructs fused to the red fluorescent protein DsRed, or as multi-domain constructs without DsRed (**c**). Potential N-glycosylation sites had been removed by replacing relevant asparagine residues by alanine in the first EGF-like domain of MSP1, the second EGF-like domain of MSP10 and in *Pfs*25. SAR: scaffold attachment region; CaMV 35S promoter and terminator: promoter with duplicated enhancer and terminator of the Cauliflower mosaic virus (CaMV) 35S gene; 5′-untranslated region: 5′-UTR of the chalcone synthase gene from *Petroselinum crispum*; signal peptide sequence: transit peptide sequence of the murine antibody 24 heavy chain; *GOI* Gene of interest.

The recombinant DsRed-fusion proteins featuring EGF-like domains 1 and 2 of MSP10 as well as EGF-like domains from related merozoite surface proteins MSP4 and MSP8 used in this study were produced transiently in *Nicotiana benthamiana* as previously described [19]. Additionally, two multidomain proteins were produced in a similar way, both containing the first EGF-like domain of MSP1_19_ followed by the above-mentioned set of EGF-like domains of merozoite proteins (multi-EGF). The second multidomain molecule additionally included two blood-stage transcending proteins, Pfs25 from the sexual stage and the TSR domain of CSP as a pre-erythrocytic stage component (Figure 1).

### Ethics statement and blood donors

Ethical approval for this study was obtained from the Committee on Human Research Publication and Ethics (CHRPE) of the Kwame Nkrumah University of Science and Technology. Written informed consent was obtained from all participants after the goals of the study had been carefully explained. By the time of donation, the 31 volunteers were between 20 and 45 years old and had not had any reported malaria disease for 2 years. From each volunteer, 50 ml of heparinized blood was drawn and prepared as described below.

### Preparation of peripheral blood monocytes (PBMCs) from Ghanaian donors

PBMCs were prepared from 50 ml of heparinized peripheral blood from each of the 31 donors. The blood was diluted in 1 volume of PBS before being layered onto Ficoll (GE Healthcare, Uppsala, Sweden). After centrifugation (800×*g*, 20 min, 25°C) the PBMCs in the interphase were harvested, washed once in PBS containing 1% (v/v) FCS (400×*g*, 10 min, 25°C) and once in PBS containing 10% (v/v) FCS (300×*g*, 10 min, 25°C) before being cryopreserved in freezing medium (10% (v/v) DMSO in FCS) at cellular concentrations ranging from 3 to 6 × 10^6^ cells/ml. The temperature was gradually decreased to −80°C (1°C/min) using isopropyl-containing freezing containers.

### ELISA

The reactivity of Ghanaian plasma samples and antibodies in supernatants of lymphoblastoid cell lines (LCLs) against MSP10 was assessed by ELISA as described [20]. An amount of 100 ng of the respective antigen was coated in wells of high-binding ELISA plates overnight at 4°C. Wells were washed three times with 0.1% (v/v) Tween20 in PBS (137 mM NaCl, 2.7 mM KCl, 8.1 mM Na_2_HPO_4_, 1.5 mM KH_2_PO_4_; pH 7.4) and then blocked with 200 µl 2% (w/v) milk powder in PBS for 2–3 h at 37°C. Following another three washing steps, 50 µl of each supernatant or diluted plasma sample was transferred to each well. As positive control, a dilution series of a mixture of four Ghanaian plasma samples was used. Plasma from European malaria-naïve blood donors served as negative control. Plates were then incubated at 37°C for 1 h. After washing three times, goat-anti-human IgG conjugated to alkaline phosphatase (AP) (Dianova, Hamburg, Germany) in PBS (1:5,000) was added. After an incubation step at 37°C for 1 h and three washing steps, *p*-nitrophenyl phosphate was added. Absorbance at 405 nm was measured after 20–45 min. Based on the reactivity of the positive control, a standard curve was fitted with a four-parameter logistic model, using the open-source software “R” for statistical computing [21].

### Fluorescent labelling of antigens

For sorting of antigen-specific B cells, the multidomain protein E25T was conjugated to Alexa Fluor^®^ 488 using the “Alexa Fluor^®^ 488 5-TFP” kit (Life Technologies, Darmstadt, Germany). Labelling efficiency was checked according to the manufacturer’s instruction by absorbance measurements at 494 nm (Alexa Fluor^®^ 488 concentration) and 280 nm.

### Reduction and alkylation of recombinant plasmodial proteins

Recombinantly expressed plasmodial proteins were reduced in 5 mM DTT at 56°C for 45 min while shaking. The reactions were stopped by adding 15 mM iodoacetamide. After incubation for 30 min in the dark, 5 mM DTT were added to quench excessive iodoacetamide.

### Generation of Epstein-Barr virus (EBV)-containing supernatants from B95-8 cells

Marmoset monkey lymphocytes B95-8 (#ACC100, DSMZ, Berlin, Germany) were used to generate preparations of infectious EBV. To this end, cells were seeded at a density of 10^6^ cells/ml and kept in RPMI 1640 with Glutamax™, 100 U/ml penicillin, 100 μg/ml streptomycin, 10% (v/v) FCS for 4 to 11 days. After centrifugation (800×*g*, 10 min, 25°C) supernatants were passed through sterile 0.45 μm-filters, snap frozen in liquid nitrogen and stored at −80°C.

### Flow cytometric cell sorting

PBMCs from a semi-immune donor were thawed gently, washed once in RPMI 1640 containing 10% (v/v) FCS and subjected to a staining procedure with a sterile mixture of AlexaFluor^®^ 488-labelled antigen (E25T), anti-human IgG-RPE (BD Biosciences, Heidelberg, Germany) and anti-human CD22-APC (BD Biosciences) in labelling buffer (sterile PBS without MgCl_2_ and CaCl_2_ containing 2% (v/v) FCS and 1 mM EDTA, pH 7.4) at 4°C for 30 min in the dark. Following three washing steps in ice-cold labelling buffer cells were filtered through a 33 µm-mesh, taken up in particle-free RPMI 1640 containing 10% (v/v) FCS. CD22^+^/IgG^+^ antigen-specific mature B cells were sorted with a FACSAriaII cell sorter (BD Biosciences) and collected in RPMI 1640 containing 20% (v/v) FCS.

### EBV transformation of antigen-specific B cells

B cells selected by cell sorting were then transformed as described before with minor modifications [22]. B cells were taken up in B cell medium (RPMI 1640 medium supplemented with l-glutamine, 10 mM HEPES buffer, 1 mM sodium pyruvate, 10% (v/v) FCS), 1 µg/ml CpG2006 and 30% (v/v) EBV-containing B95-8 supernatant (3.4 x 10^8^ viral copies/ml). An amount of 50 B cells was subsequently seeded onto 100,000 irradiated (83 gray) allogeneic PBMCs in 96 well round-bottom tissue culture plates and incubated at 37°C, 5% CO_2_. After 2 weeks, supernatants were removed completely and replaced by B cell medium containing 1 µg/ml CpG 2006 and 50 U/ml rhIL-2 (Roche, Basel, Switzerland). After another week, supernatants were tested for secretion of total and specific IgG.

### Dotblot

Dotblots were used to rapidly assess secretion of IgG by LCLs and to analyse the nature of MSP10 epitopes. In order to check for secreted total human IgG, 3 µl of the culture supernatants were transferred onto a nitrocellulose membrane (Roth, Karlsruhe, Germany) and allowed to dry for at least 15 min. As positive control, a dilution series of a mixture of four Ghanaian plasma samples was used ranging from 1:100 to 1:3,200. Blocking was carried out in 5% (w/v) non-fat milk powder in PBS for 30 min at 25°C. Subsequently, the blocking buffer was substituted by a 1:5,000 dilution of goat-anti-human IgG-AP in 5% (w/v) non-fat milk powder in PBS. Following incubation for 1 h, the membrane was washed thoroughly with 0.05% (v/v) Triton X-100 in PBS. Detection was carried out with NBT/BCIP. To determine whether conformational or linear epitopes of the antigens are recognized by the antibodies, native and reduced/alkylated antigens were applied onto nitrocellulose membranes.

### Spectratyping

For the characterization of the LCL clonality, genomic DNA (gDNA) and RNA were used. The PCR conditions and the primers used have been described elsewhere [22]. As a monoclonal control gDNA from Ramos cells was used; gDNA from PBMCs served as a polyclonal control sample. Amplicons were analysed with a 3730 DNA Analyser (Applied Biosystems/Life Technologies).

### Rescue of human immunoglobulin variable regions

RNA and gDNA were prepared either using the “NucleoSpin Tissue” and “NucleoSpin RNA II” kits (Macherey–Nagel, Düren, Germany) or the “RNeasy Plus Mini” kit (Qiagen, Hilden, Germany). Messenger RNA (mRNA) from LCLs was reverse transcribed using the “SuperScript III” kit (Life Technologies). Human heavy chain and light chain variable regions (Vh and Vl regions) from LCLs were recovered as described before, with minor modifications [23]. Our approach differed in that mRNA from B cell clusters instead of from single cells was used. Moreover, (1) a re-amplification step with the primer set for the first PCRs was included and (2) these sequences were cloned into a TopoVector (pCR^®^II-TOPO^®^, Life Technologies) in order to be able to remove potential endogenous cleavage sites. All kits were used according to the manufacturers’ instructions.

### Bioinformatic analysis of human immunoglobulin variable regions

Variable regions were analysed in silico by the IMGT/V-QUEST algorithm in order to determine the nature of the assembled V-, D- and J-gene segments and the germline homology [24]. The identity and similarity of the obtained Vh and Vl regions compared to their most closely related underlying germline sequences was determined by independently analyzing each of the underlying gene segments by an identity/similarity tool [25]. The respective complementarity determining regions (CDRs) were identified according to the Kabat rules [26]. Alignments were done with Clustal Omega [27]. Potential sulfation of tyrosine residues was predicted by the Sulfinator algorithm [28].

### Cloning

All enzymes for cloning purposes were obtained from NEB (Frankfurt am Main, Germany). Amplicons from V regions were cloned into pTRAkt plant expression vectors. To reconstitute full-size heavy (IgG1) or light (Igκ) chain genes, amplicons of the Vh region sequences from the second V region-PCR were cloned into a suitable pTRAkt vector in frame with a sequence encoding a human IgG1 constant domain via *Age*I/*Sal*I; Vl-region sequences were cloned via *Age*I and *BsiW*I *in frame* with a sequence encoding a human Igκ constant domain as previously described [29]. The resulting pTRAkt vectors were used for transformation of *Agrobacterium tumefaciens* (*Rhizobium radiobacter*) and subsequent transient expression of humAbs in *Nicotiana benthamiana* plants.

### Transient expression of recombinant human full-size IgG in *Nicotiana benthamiana*

Electro-competent agrobacteria (*Agrobacterium tumefaciens* strain GV3101: pMP90RK with resistance to gentamycin, kanamycin and rifampicin) were transformed with one of the following pTRA vectors: pTRAc-p19si, pTRAkt_H_5E8, pTRAkt_H_5F6, pTRAkt_κ_5E8, or pTRAkt_κ_5F6 as previously described [19, 30]. Plants were 8–10 weeks old by the time of infiltration and had been raised in rock wool in a phytotron as described previously [30]. Plants were immersed into the agrobacterial suspension, infiltrated for 1 min at 5 kPa, 25°C and then cultivated for 5 days with steady light and regular watering, i.e. spraying for 1 min every 3 h. After 5 days of incubation the infiltrated plants were harvested. Leaves were shredded in two to three volumes of extraction buffer (PBS containing 10 mM Na_2_SO_5_; pH 7.4, 4°C) for 1 min. Insoluble material and plant fibers were removed by filtration through Miracloth. Then, 500 mM NaCl was added, pH was adjusted to 8.2 and the suspension was incubated at 4°C for 1 h. Precipitated material was then removed by centrifugation (17,000×*g*, 30 min, 4°C) and a successive filtration step using 0.45 µm-filters.

### Antibody purification by Protein A chromatography

HumAbs were purified from *Nicotiana benthamiana* extracts as well as lymphoblastoid cell culture supernatants by Protein A chromatography using MabSelect™ SuRe™ Sepharose (GE Healthcare). The purification matrix was equilibrated with binding/washing buffer (0.2 M Tris–HCl, pH 9.0) prior to the affinity chromatography. Antibodies were eluted with 0.2 M Sodium Citrate, pH 2.5, elution fractions were neutralized by 1 M Tris, pH 9.0 and dialyzed against PBS. For use in in vitro growth inhibition assays (GIAs) antibodies were concentrated in RPMI 1640 incl. 25 mM HEPES (PAA, E15-041), using Vivaspin 15R 30,000 MWCO centrifugal concentrators (VWR 512-4124) and sterilized by filtration.

### *Plasmodium falciparum* culture

*Plasmodiumfalciparum* strain 3D7A was cultured as described before [31]. Briefly, parasites were maintained at a haematocrit of 5% (v/v) in parasite culture medium [RPMI 1640, 25 mM HEPES, 2 mM l-glutamine, 50 μg/ml gentamycin, 10% (w/v) Albumax^®^II (Life Technologies)]. Erythrocytes of 12–16 individual donors, all having the blood group 0, Rh-positive, were obtained in CPDA tubes from the Department for Transfusion Medicine, RWTH Aachen University Clinic, Aachen, Germany. The suspensions were mixed, centrifuged in order to remove plasma and white blood cells (650×*g*, 5 min, 25°C, without break) and washed for three times in SAG-mannitol (150 mM NaCl, 50 mM d-glucose, 1.2 mM adenine, 28.8 mM D(-)-mannitol). Washed erythrocytes were taken up in SAG-mannitol to obtain a final haematocrit of 66.6% (v/v).

### In vitro growth inhibition assay (GIA)

In vitro growth inhibition of the isolated antibodies was assessed via GIA on *P.falciparum* as described before using humAbs in final concentrations ranging from 10 to 0.078 mg/ml in RPMI 1640 containing 25 mM HEPES [32]. As positive control, 6 mg/ml polyclonal rabbit-anti-AMA1 IgG (BG98) was used [33]. As corresponding negative control 6 mg/ml polyclonal IgG of a naïve rabbit were used. The humAb 2G12, an HIV-1-specific IgG, was taken as a plant-expressed non-specific control [34]. *P.falciparum* 3D7A were synchronized by three sorbitol treatments 7, 5 and 3 days prior to the GIA [35]. Schizont stage parasites were seeded at 0.3 or 0.4% parasitaemia in 2% (v/v) haematocrit. Each GIA was performed with 50 μl final volume in 96-well flat-bottom half-area culture plates. After an incubation period of 44 h, GIAs were harvested and frozen at -20°C for at least 16 h. Parasite growth was estimated by a pLDH-assay [36]. The percentage of relative growth inhibition was calculated as follows: % inhibition = 100 × [A_655 nm_ (sample) − A_655 nm_ (erythrocyte control)]/[A_655 nm_ (schizont growth control) − A_655 nm_ (erythrocyte control)].

### Immunofluorescence assay

Synchronous *P.falciparum* 3D7A were used to prepare IFA slides. Smeared parasites were fixed in methanol at −20°C for 10 min. After blocking in 1% (v/v) FCS (low IgG) in PBS for 3 h at 25°C, slides were incubated with 50 µg/ml mouse-anti-MSP4 and either of the three human-anti-MSP10 antibodies (10 or 25 µg/ml) or 2G12 (an anti-gp 120 HIV-1 humAb as isotype control for human IgG) in 1% (v/v) FCS (low IgG), 0.01% (w/v) Saponin in PBS) in a moist box at 25°C for 1 h. After four washing steps in PBS, the slides were treated with 1:100 goat-anti-mouse IgG(H + L)-Alexa Fluor^®^ 488 (Life Technologies), 1:1,000 goat-anti-human IgG(H + L)-Cy3 (Dianova) and 10 µg/ml Hoechst 33342 (Life Technologies) in a moist box at 25°C for 1 h. Five washing steps were carried out. Prolong^®^ Antifade mounting medium (Life technologies) was used to conserve the staining. Images were taken at a 630× magnification with a Leica TCS SP8 microscope and LAS AF Version 3.1.3 software (Leica, Solms, Germany).

### Surface plasmon resonance (SPR) spectroscopy

Kinetic binding parameters of the monoclonal antibodies were analysed by SPR spectroscopy using a Biacore T200 instrument (Biacore, GE Healthcare, Uppsala, Sweden) and Protein A-functionalized CM5-S series sensor chips as previously described [29]. For kinetic analysis, 100–180 RU of the different humAbs were captured onto immobilized protein A and recombinant multi-EGF (0.1–540 nM) was injected at a flow rate of 30 µl/min for 180 s. Dissociation was tracked for 600 s. Between measurements, the surface was regenerated by pulsing for 1 min with 30 mM HCl. Buffer injections were used for double referencing. All measurements were performed at 25°C using HBS-EP (10 mM Hepes, 150 mM NaCl, 3.4 mM EDTA, 0.005% (v/v) Tween-20) as running buffer. Binding curves were evaluated using BIAEval 4.0 (GE Healthcare).

## Results

### Reactivity of plasma from Ghanaian semi-immune donors against MSP10 EGF-like domains

The study was designed to efficiently isolate humAbs which recognize the EGF-like domains of MSP10 and which represent a part of the naturally-acquired, protective immunoglobulin repertoire of semi-immune donors. The definition of semi-immunity of the plasma donors was primarily based on observation that they had not shown any signs of clinical malaria for 2 years. Moreover, several screenings had been performed to make sure that the set of donors had a broad anti-malarial antibody response, as published before [13, 19, 30, 37–39].

**Figure 2:**
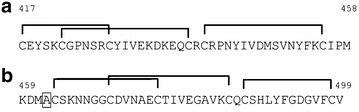
Epidermal growth factor (EGF)-like domains of MSP10. The sequences of the EGF-like domains one (**a**) and two (**b**) of MSP10 from the *Plasmodium falciparum* parasite strain 3D7A are shown. Connecting *lines* between the cysteine residues represent the disulfide bridges. Sequences were derived from http://www.uniprot.org/uniprot, accession number C6HT44. An asparagine residue was replaced by an alanine (N462A, *boxed*) in order to prevent N-glycosylation in plants.

**Figure 3:**
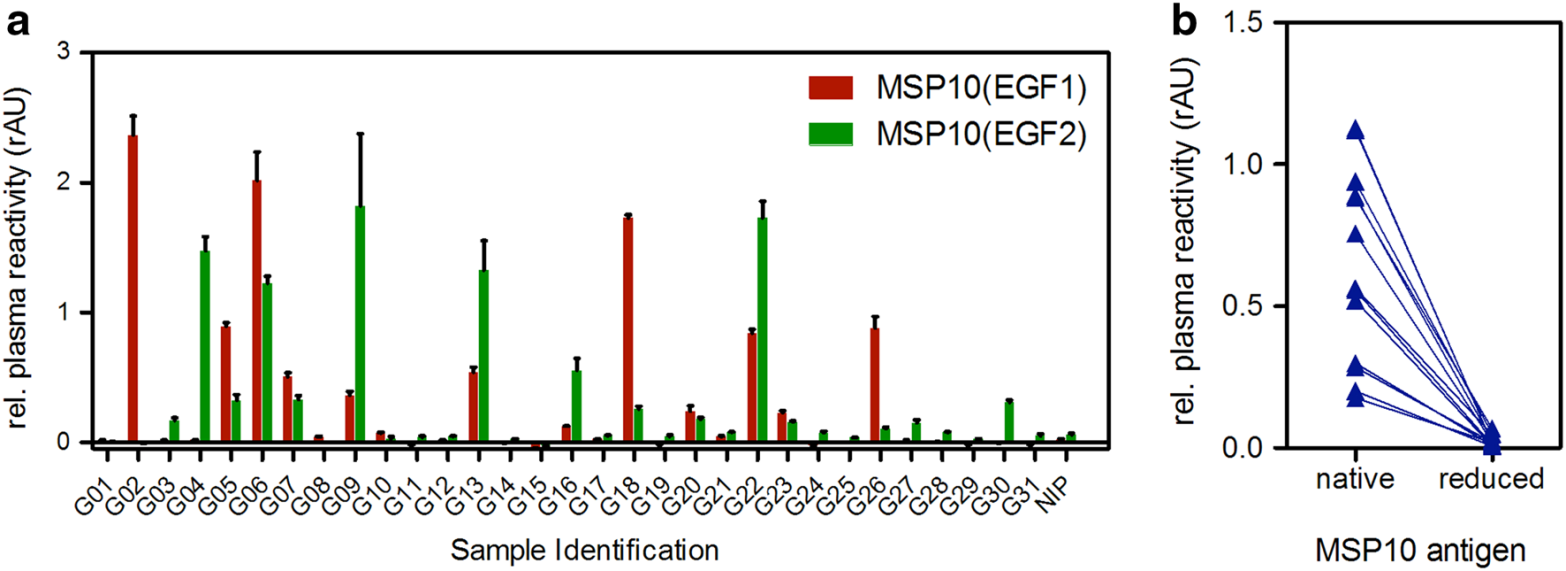
Reactivity of Ghanaian plasma samples against both EGF-like domains of MSP10. **a** Reactivity of 31 Ghanaian plasma samples and a European non-immune control plasma pool (NIP) were tested against the first (*red bars*) or second (*green bars*) EGF-like domain of MSP10 by ELISA. Reactivities were measured against the DsRed-MSP10(EGF) fusion protein. Thus, the background reactivity against DsRed, which was very low in all samples (absorbance at 405 nm below 0.1), was subtracted from all samples. Values represent relative reactivities in comparison to the positive control pool, whose reactivity was set to “1”. **b** Plasma samples positively reacting to MSP10 were also tested for reactivity against the reduced and alkylated antigen multi-EGF. All data were measured in technical triplicates. *Error bars* represent the standard deviation of sample results.

To quantify the MSP10-response, plasma of 31 Ghanaian blood donors were screened for reactivity against DsRed-MSP10(EGF1) (Figure 2a) and DsRed-MSP10(EGF2) (Figure 2b). In order to exclude background binding of the plasma samples against the fusion partner DsRed, background reactivity against DsRed was measured as well and subtracted from the reactivity against the respective fusion protein (Figure 3a). In this ELISA, 15 plasma samples (48%) showed specific reactivity (>reactivity of negative control + 2 × standard deviations) against DsRed-MSP10(EGF1); 15 (48%) showed specific reactivity against DsRed-MSP10(EGF2). Interestingly, eleven of these recognized both EGF-like domains of MSP10. Therefore, B cells from these individuals appeared to be a suitable source of pan-MSP10 EGF-like domain humAbs.

It had been reported that naturally acquired antibodies which are specific for plasmodial EGF-like domains primarily react with conformational epitopes [17, 40, 41]. Thus, these data were especially confirmed for MSP10. To this end, native as well as reduced and alkylated form of the multi-EGF fusion protein, containing both EGF-like domains of MSP10, was used for an ELISA with 13 of the Ghanaian plasma samples which were positive for either or both EGF-like domains of MSP10 (Figure 3b). The reactivity of each of the semi-immune plasma samples was reduced to background level when the antigen had been reduced and/or alkylated demonstrating that IgG of the semi-immune donors specifically recognize conformational epitopes of the MSP10 EGF-like domains.

### Selection and EBV transformation of mature IgG^+^ antigen-specific B cells

For selection of mature, IgG^+^, merozoite surface antigen-specific B cells as basis for the subsequent EBV transformation, PBMCs from donor G13, one of the donors whose plasma reacted with both of the MSP10 EGF-like domains, were used. An amount of 1.25 × 10^7^ vital PBMCs was subjected to flow cytometric sorting and 4,869 vital CD22^+^/IgG^+^ B cells (0.039%) were selected and seeded for EBV transformation. After 7 weeks, 6 out of 90 wells (6.67%) showed reactivity with the multi-domain fusion protein E25T, which includes both EGF-like domains of MSP10. The corresponding LCLs were expanded. Fifteen weeks after EBV transformation four of these six cultures had ceased to secrete specific IgG. The remaining two cultures, 5E8 and 5F6, were used for clonal analyses by spectratyping and rescue of Vh and Vl sequences by PCR.

Spectratyping revealed that LCL 5F6 was monoclonal as early as 16 weeks post infection. At that time point LCL 5E8 was still octaclonal. After continuous subculturing of the LCL 5E8 for another 21 weeks, it also became monoclonal.

### Rescue and analysis of antibody variable sequences

**Figure 4:**
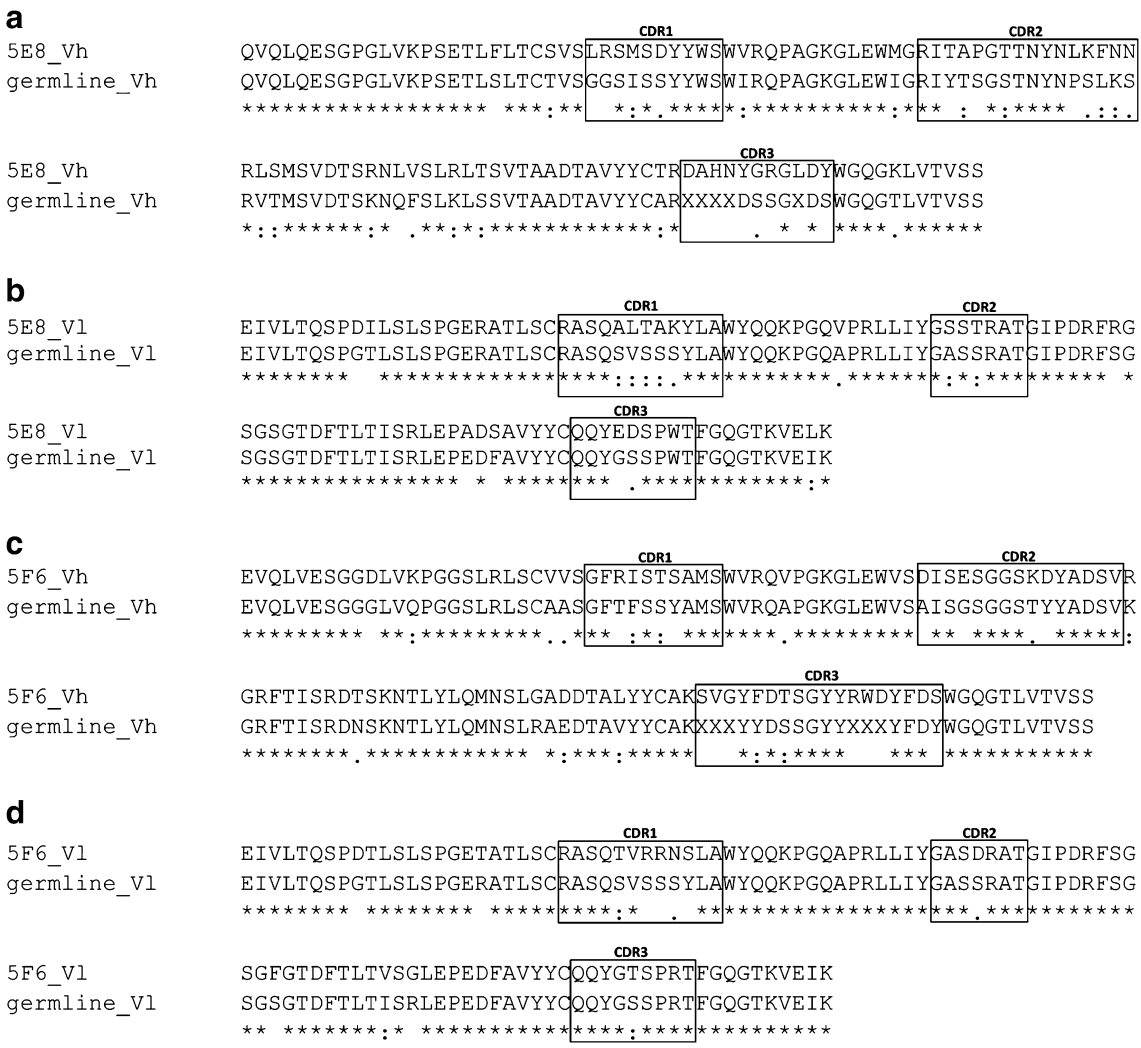
Alignment of Vh and Vl sequences of 5E8 and 5F6 with their respective germline sequence. The amino acid sequences of the obtained human antibody V region sequences rescued from LCLs 5E8 (**a**, **b**) and 5F6 (**c**, **d**) were aligned to their respective germline sequence, as obtained by the IMGT/V-Quest Tool [24]. Joining region elements containing N-nucleotides between the V-, D and J-segments for the Vh (**a**, **c**) are marked by the undefined amino acid X. CDR1-3 (*boxes*) are labelled according to the Kabat definition.

Each of the LCLs 5E8 and 5F6 were subjected to RNA isolation, cDNA synthesis and amplification of immunoglobulin Vh-, Vk- and Vl- region sequences. From each culture, one Vh and one Vk were recovered. Subsequently, the Vh and Vl regions were sequenced and compared to the closest corresponding germline sequences. As shown in Figure 4, 60 DNA mutations in the Vh 5E8 sequence and >40 DNA mutations in the Vl 5E8 sequence led to 31 and 16 amino acid changes, respectively (Figure 4a, b). Sequence alignments for Vh 5F6 (Figure 4c) and Vl 5F6 (Figure 4d) revealed 21 changes (≥45 DNA mutations) and 12 changes (≥26 DNA mutations), respectively.

### Recombinant expression of humAbs

Variable region sequences of 5E8 and 5F6 were cloned into pTRAkt vectors for recombinant transient expression in *Nicotiana benthamiana*. Since antibody heavy and light chains are encoded on separate pTRAkt plant expression plasmids it is possible to realize any HC/LC combination. After initial small scale expression studies three (H5F6:κ5E8, H5F6:κ5F6 and H5E8:κ5E8) of the four possible Vh/Vl combinations recognized MSP10. Subsequently, the three corresponding binding-competent humAbs were transiently expressed at a medium scale (10-16 *Nicotiana benthamiana* plants). Heavy chain 5F6 (H5F6) was either co-expressed with the light chain 5E8 (κ5E8) or 5F6 (κ5F6), respectively, resulting in humAb10.1 (H5F6:κ5E8) and humAb10.2 (H5F6:κ5F6). Heavy chain 5E8 (H5E8) was co-expressed with its natural light chain (κ5E8), thus forming humAb10.3 (H5E8:κ5E8). The average yield of the recombinant antibodies was 100-200 mg per kg of fresh leaf material.

### Specific binding comparison between antibodies from LCL supernatants and respective recombinant humAbs

**Figure 5:**
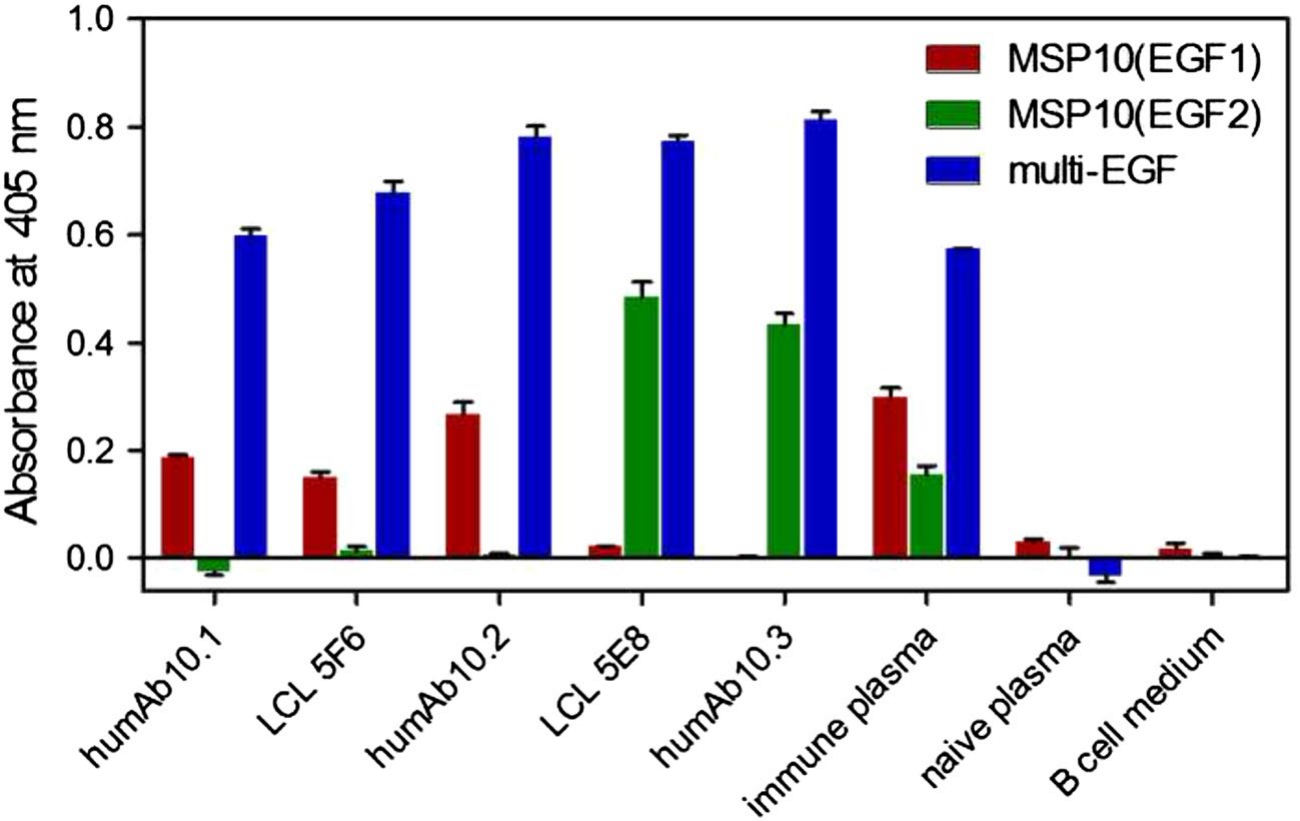
Specific reactivity of antibodies produced by the LCLs versus their derived recombinant humAbs. Using ELISA, the specific reactivity of the antibodies from the supernatant of LCLs (undiluted, three-day culture) as well as the derived recombinant humAbs transiently produced in *Nicotiana benthamiana* against the first (*red bars*) or second (*green bars*) EGF-like domain of MSP10 and multi-EGF containing a series of plasmodial EGF-like domains (blue bars). Represented values reflect the reactivity against the fusion proteins corrected by the reactivity observed with the his-tagged control protein (DsRed). Recombinantly expressed and purified humAb10.1 (H5F6:κ5E8), humAb10.2 (H5F6:κ5F6) and humAb10.3 (H5E8:κ5E8) were applied in concentrations of 150, 1.5 and 3 µg/ml, respectively. A pool of Ghanaian plasma samples at a dilution of 1:200 served as positive control, a pool of naïve plasma samples as negative control. Plain B cell medium was used as negative control for the LCL. The ELISA was performed in technical triplicates; the *error bars* represent the standard deviations.

The reactivity of the humAbs against MSP10(EGF1) and MSP10(EGF2) was verified by ELISA with DsRed-MSP10(EGF1), DsRed-MSP10(EGF2) and multi-EGF comprising both EGF-like domains of MSP10. All humAbs bound to multi-EGF (Figure 5). The recombinant humAb10.3 and the homologous LCL 5E8 supernatants similarly reacted with MSP10(EGF2), but not MSP10(EGF1). Likewise and complementarily, LCL-derived IgG 5F6 and recombinant humAb10.2 recognized MSP10(EGF1), but not MSP10(EGF2). The shuffled antibody humAb10.1, which by definition could only be recombinantly expressed, recognized the first EGF-like domain of MSP10 and thus showed the same specificity as humAb10.2.

The affinity of the humAbs to MSP10 was analysed by SPR spectroscopy using the multi-EGF fusion protein, which is monovalent and contains several EGF-like domains of the MSP family including both EGF-like domains of MSP10. Kinetic constants k_a_, k_d_ and K_D_ of both humAb10.2 and humAb10.3, produced in different expression systems, were determined. The K_D_ values of all antibodies are summarized in Table 1. High association rates (k_a_) for both humAb10.2 and humAb10.3 were observed. However, the dissociation rate (k_d_) of humAb10.3 was lower than the one of humAb10.2, indicating that humAb10.3 possesses a higher affinity for MSP10(EGF2) than humAb10.2 for MSP10(EGF1). The affinity of the humAb10.1, which is composed of the artificial combination of H5F6:κ5E8, to its antigen MSP10(EGF1) was more than 100-fold lower than the affinity of humAb10.2 for the same antigen. The recombinant humAb10.2 and the corresponding LCL-derived 5F6 antibody showed a comparable affinity to the antigen MSP10(EGF1). Surprisingly, the affinity of the humAb10.3 expressed in plants differs by a factor of seven from the respective antibody produced by the corresponding LCL (5E8).

**Table 1:** Affinities (K_D_ values) of purified humAb10.1, humAb10.2 and humAb10.3 as determined by SPR spectroscopy using the recombinant MSP-based fusion protein multi-EGF

Antibody	Expression system	K_a_ (M^−1^ s^−1^)	K_d_ (s^−1^)	K_D_ (M)
humAb10.1 (H5F6:κ5E8)	*Nicotiana benthamiana*	4.43 × 10^3^	4.11 × 10^−3^	9.27 × 10^−7^
humAb10.2 (H5F6:κ5F8)	*Nicotiana benthamiana*	5.31 × 10^5^	2.90 × 10^−3^	5.46 × 10^−9^
	LCL 5F6	4.46 × 10^5^	2.95 × 10^−3^	6.62 × 10^−9^
humAb10.3 (H5E6:κ5E8)	*Nicotiana benthamiana*	2.32 × 10^5^	1.15 × 10^−3^	5.39 × 10^−9^
	LCL 5E8	3.99 × 10^5^	3.06 × 10^−4^	8.04 × 10^−10^

A potential explanation for this difference might be a posttranslational modification of the antibody, e.g. the sulfation of tyrosine residues, which is assumed not to take place in plants [42]. Therefore, in silico analysis was performed using the Sulfinator tool [28]. Two residues in the CDR1 (LRSMSDYY) and one residue in the CDR3 (DSAVYYCQQYED) of the heavy chain of humAb10.3 were predicted to be sulfated in mammalian expression systems.

### Recognition of conformational epitopes of humAb10.1, humAb10.2 and humAb10.3

Knowing that antibodies of the protective immunoglobulin repertoire of semi-immune individuals mainly recognize correctly folded plasmodial EGF-like domains as shown in Figure 3b and by others [17, 40, 41], antibodies humAb10.1, humAb10.2 and humAb10.3 were tested for their binding capacities against conformational epitopes of different members of the merozoite surface protein family and MSP10. Analysis by dotblots showed that all three humAbs bound conformational epitopes of MSP10(EGF1) or MSP10(EGF2), respectively, but did not bind to their reduced counterparts. Moreover, they did not cross-react with other plasmodial EGF-like domains of MSP1, MSP4 or MSP8.

**Figure 6:**
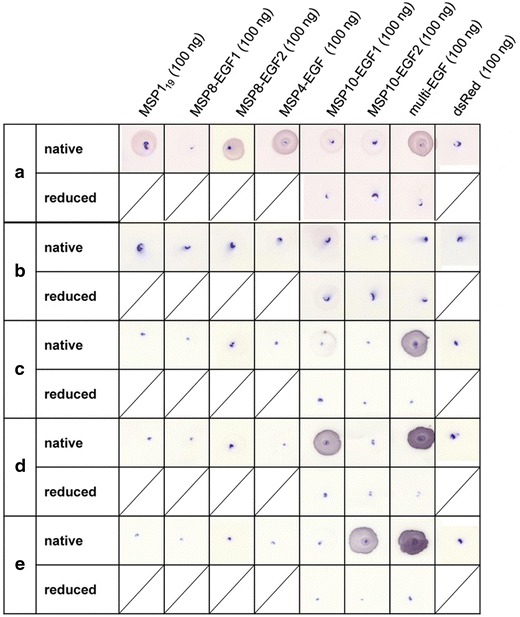
Dotblots with native and reduced EGF-like domains of *P. falciparum* merozoite surface proteins. Native proteins MSP1_19_, DsRed-fusion proteins of MSP8(EGF1), MSP8(EGF2), MSP4(EGF), MSP10(EGF1) and MSP10(EGF2), multi-EGF and DsRed were applied in the indicated amounts. Proteins containing any of the EGF-like domains of MSP10 were also reduced and alkylated and used in the same quantity. The Ghanaian plasma pool (**a**) and the naïve plasma pool (**b**) were used in a 1:500 dilution. Recombinantly expressed and purified humAb10.1 (**c**), humAb10.2 (**d**) and humAb10.3 (**e**) were used in concentrations of 100, 50 and 1.4 µg/ml, respectively. As secondary antibody, goat-anti-human IgG-AP (Dianova) at a dilution of 1:5,000 was used.

In conclusion, all human antibodies described here only bind the native antigen, thus recognizing a conformational epitope. Secondly, all three antibodies bind highly specific to MSP10 EGF-like domains, and do not show any cross-reactivity (Figure 6).

### IFA of the anti-MSP10 humAbs

**Figure 7:**
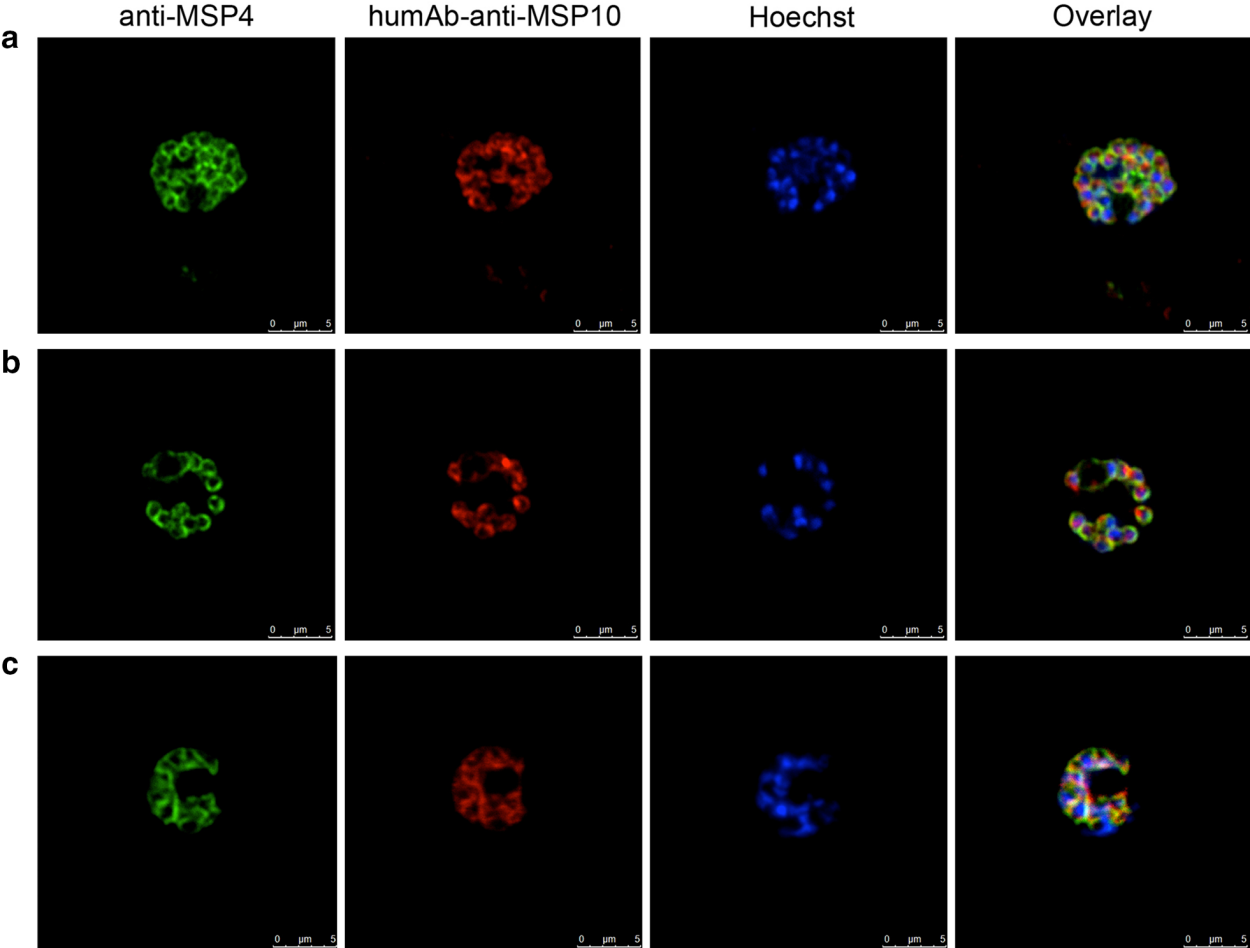
IFAs of recombinantly expressed humAbs *P.* *falciparum* 3D7A schizonts. Binding of humAb10.1, humAb10.2 and humAb10.3 to schizonts was assessed by IFA. Parasites were co-stained with a mouse-anti-MSP4 antibody (*green*), Hoechst 33342 nuclear stain (*blue*) and either of the three recombinant humAb10.1 (**a**), humAb10.2 (**b**) and humAb10.3 (**c**) (*red*). Human and murine antibodies were detected by fluorescently labelled antibodies goat-anti-human IgG (H + L)-Cy3 (Dianova) and goat-anti-mouse IgG (H + L)-Alexa Fluor^®^ 488 (Life Technologies), respectively. Images were taken at a 630× magnification with a Leica TCS SP8 microscope. The *right panel* shows the overlay of the three fluorescence images. *Scale bar* 5 µm

In order to validate, if the antibodies not only recognize recombinantly produced EGF-like domains of MSP10, but also naturally expressed MSP10, we performed immunofluorescence assays. To this end, segmented *P.falciparum* 3D7A schizonts were used for confocal microscopy with humAb10.1, humAb10.2 and humAb10.3 (Figure 7). As counterstain, a murine monoclonal MSP4(EGF)-specific antibody was used [29]. As an isotype control of human IgG, the anti-gp 120 HIV-1 humAb 2G12 instead of the MSP10-specific humAb was used. No signal was detected in this setup. HumAb10.1 (Figure 7a), humAb10.2 (Figure 7b) and humAb10.3 (Figure 7c) showed partial co-localization with the anti-MSP4 antibody. Additionally, the humAbs recognized a dot-like structure at the apical end of the preformed merozoites in the schizont stage.

### Inhibition of *P. falciparum* 3D7A growth by MSP10-specific humAbs in vitro

**Figure 8:**
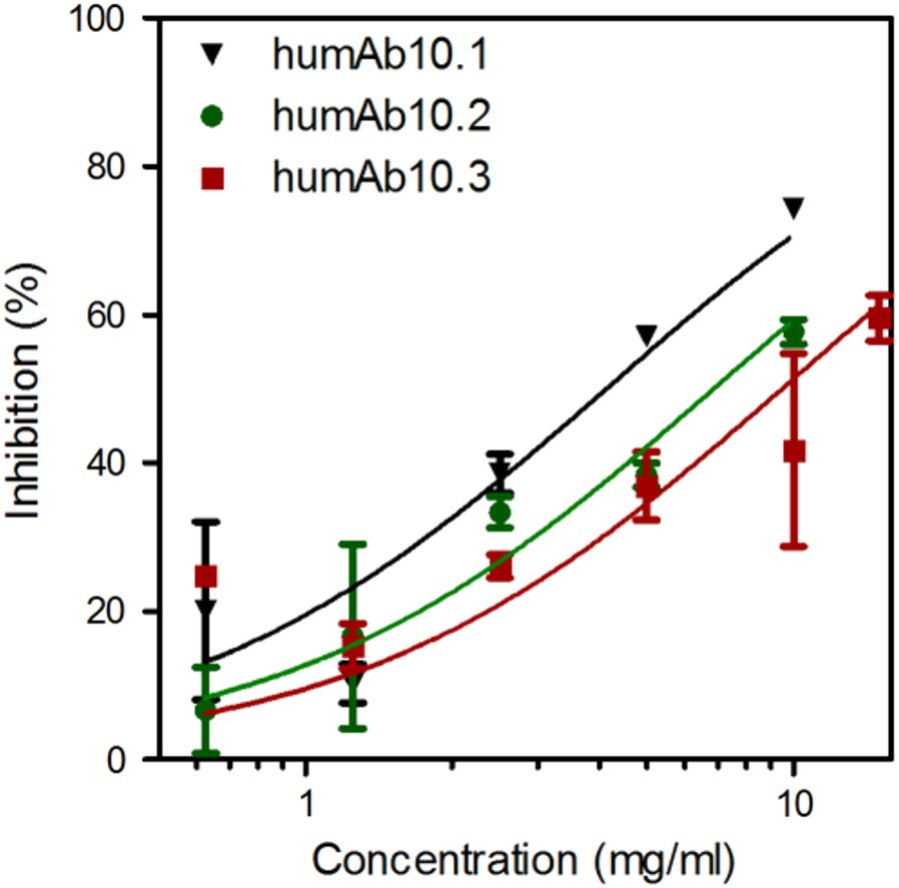
In vitro GIA of *P.* *falciparum* 3D7A. *Plasmodium* *falciparum* 3D7A had been synchronized three times prior to the start of the GIA at schizont stage. Recombinant humAb10.1 (*black triangles*), humAb10.2 (*green circles*) and humAb10.3 (*red squares*) have been purified by Protein A chromatography, concentrated, re-buffered in culture medium, sterilized by filtration and were applied in final concentrations of up to 10 mg/ml. The plates were harvested after 44 h and the growth of parasites estimated by a pLDH assay. All tests were carried out in technical triplicates; *error bars* correspond to standard deviation values.

In order to test the inhibitory potential of humAb10.1, humAb10.2 and humAb10.3, an in vitro-GIA with *P.falciparum* strain 3D7A was performed. All three antibodies showed a concentration-dependent inhibition of *P.falciparum* 3D7A (Figure 8). However, an inhibition of 100% was not reached by any of the antibodies at the applied concentrations; the maximum inhibition of all antibodies ranged between 60 and 70%. HumAb10.1 inhibited *P.falciparum* 3D7A most efficiently with an EC_50_ value of 4.1 mg/ml (95% confidence interval (CI) 2.6–6.6 mg/ml), humAb10.2 inhibited the parasite growth with an EC_50_ value of 6.9 mg/ml (CI 5.5–8.6 mg/ml) and humAb10.3 inhibited the merozoite invasion with at an EC_50_ value of 9.5 mg/ml (CI 5.5–16.4 mg/ml).

## Discussion

So far, only a handful of anti-plasmodial humAbs have been isolated and described. Some of these are specific for the MSPs (MSP1, MSP2 and MSP3) or GLURP [43–46]. Others show specificity for the plasmodial proteins expressed on the erythrocyte surface, such as VAR2CSA, RESA and the *Plasmodium vivax* DBP [47–49]. Another work also describes humAbs specific for the sexual stage of the parasite (Pfs48/45) or the sporozoite stage ((NPNA)_3_-repeat of CSP) [50, 51].

Here, additional human antibodies specific for the merozoite surface, isolated by an innovative human antibody technology platform, are presented. This technology combines the screening of naturally exposed semi-immune individuals from endemic countries for antigen-specific reactivity, the targeted selection of antigen-specific B cells by flow cytometric sorting, the subsequent EBV transformation, rescue of the antibody V region sequences and subsequent recombinant production in plants. This allows to produce different versions of the antibody, the antibodies produced directly in the LCLs, as well as the recombinant antibody. Combinations of different heavy and light chains can only be produced using recombinant antibodies. Moreover, the slight difference in affinity of humAb10.3, which likely relies on posttranslational modifications, can only be detected when comparing multiple expression systems.

The rescue of the antibody variable sequences is especially important, as LCLs tend to cease to secrete antibodies or stop to proliferate altogether, which would lead to an irreversible loss of the desired immunoglobulin [52]. In contrast, the transient antibody production in plants allows for rapid and scalable production, ranging from the infiltration of individual leaves to multiple plants by vacuum infiltration [29, 34]. The humAbs described here were produced in this transient production system and showed a yield of more than 100 mg per kg of plant material. This is in the range of very well producing antibodies which have previously been described [53, 54].

The first step of the procedure, the screening of plasma for specific antigen-binding, provides valuable information: On the one hand, the positive reactivity shows that specific antibodies are present in the subjects tested; on the other hand, it validates that the recombinant antigens produced in *Nicotiana benthamiana* are correctly folded. The general reactivity of these plasma samples against various *P.falciparum* antigens has previously been shown elsewhere [19, 29, 30, 37–39]. Regarding the EGF-like domains of MSP10 described here, in total approx. two-thirds of the tested plasma samples reacted with at least one of the two domains. In the case of the other plasmodial EGF-like domains it is also known that these are not recognized by all sera of semi-immune individuals; e.g. the double EGF-like domain of MSP1_19_ [55]. Moreover, the observation that Ghanaian plasma samples primarily reacted with discontinuous epitopes of the EGF-like domains of MSP10 is in line with findings of the first description of the antigen MSP10 [17]. Coherent thereto, the isolated antibodies humAb10.1, humAb10.2 and humAb10.3 just bind to the native antigen and do not recognize reduced MSP10.

It was found that each of the isolated antibody chains had accumulated a considerable number of coding mutations during their affinity maturation in vivo. It can be assumed that it takes 4 years to accumulate 20 mutations in the Vl and 30 mutations in the Vh sequences for a desirable affinity maturation to occur [56]. Consequently, it is likely that the in vivo-maturation of humAb10.2 (H5F6:κ5F6) and humAb10.3 (H5E8:κ5E8) took at least 2–3 years and 3–4 years, respectively. This is well in line with the observation that it takes years for a protective anti-plasmodial immunoglobulin repertoire to arise [6–8].

By ELISA and SPR spectroscopic measurements it was demonstrated that (1) the correct variable regions of LCLs 5E8 and 5F6 were rescued and that (2) the corresponding recombinant IgG1:κ full-size antibodies shared the same characteristics with their naturally produced counterparts. It appears that the correct variable sequences had been recovered, cloned and expressed in the context of IgG1:κ given the facts that (1) LCLs 5E8 and 5F6 were monoclonal by the time of the rescue of their variable immunoglobulin regions and that (2) naturally and recombinantly produced humAb10.2 and humAb10.3 showed the same respective specificities. Interestingly, the artificial combination H5F6:κ5E8 of humAb10.1 featured the same specificity for MSP10(EGF1) as humAb10.2 (H5F6:κ5F6). However, humAb10.1 was much less affine for MSP10(EGF1) than humAb10.2 (Table 1). This can likely be explained by the facts that the CDRs of both Vh and Vl regions of each B cell receptor are simultaneously selected for in vivo and that all six CDRs contribute to complementarity, specificity and affinity to the epitope. Thus, the features of a paratope formed by an artificial Vh:Vl combination are likely to be compromised and may result in loss of affinity or even specificity. The fact that humAb10.1 still recognized MSP10(EGF1) argues for a major role of the heavy chain CDRs in the recognition of MSP10(EGF1). This is in line with the finding that in many antibodies CDRH2 and CDRH3 contribute most to the free binding energy [57].

IFAs using humAb10.1, humAb10.2 and humAb10.3 showed a fluorescence pattern which closely resembles the one seen before by Black et al. [17]. The antibodies bound to the merozoite surface and marked a specific spot in the region of the apical end of the merozoite. This is consistent with a localization at the rhoptries, the micronemes or a predominant localization at the apical end of the merozoite.

The EGF-like domains of MSP10 are very conserved [17, 58, 59], which argues for a critical role that does not allow for many changes in amino acid sequence and structure. It is tempting to believe that such invariable epitopes on free merozoites exposed to the serum constitute an Achilles’ tendon for inhibitory antibodies. Nevertheless, the inhibitory concentrations of the presented humAbs are relatively high. As compared to other anti-plasmodial antibodies, such as AMA1- or Rh5-reactive IgG showing EC_50_ values of as low as 50–240 µg/ml [60, 61], the inhibition of *P.falciparum* by either MSP10-specific humAb thus appears relatively inefficient. To get a more precise idea of the general inhibitory potential of naturally-acquired antibodies against MSP10, it would be highly informative to purify antibodies against MSP10 by affinity purification and subsequently measure their inhibitory potential. Unfortunately, we were not able to perform such an experiment due to the limited availability of the respective plasma samples.

Puentes et al. found three synthetic 20-mer peptides of MSP10 to be binding to human erythrocytes and to be hampering the invasion of merozoites into erythrocytes in vitro. Interestingly, one of these peptides corresponds to a part of the first EGF-like domain of MSP10 and showed 65% (±2%) inhibition at a concentration of 200 µM (0.46 mg/ml) [18]. The EC_50_ values of the antibodies described in the present work range from 4.1 to 9.5 mg/ml. This corresponds to a molar concentration range between 27 and 63 µM. Thus, the EC_50_ values of the presented humAbs are well below the concentrations needed for the linear peptides of MSP10, but still way above the the EC_50_ values of some antibodies against AMA1 and or Rh5, as described above. The probable mechanism of the MSP10-specific humAbs is the direct blocking of the binding of the merozoite to the erythrocyte, and thereby inhibiting the attachment/invasion. In case of MSP1, antibodies directed at MSP119, the portion which is comparable to the EGF-like domains of MSP10, can be classified as inhibitory, neutral or blocking (i.e. counteracting the inhibition by other MSP1-specific antibodies) depending on their activity [62]. Given the similarity of the C-terminal parts of MSP1 and MSP10, the question remains which category applies to humAb10.1, humAb10.2. and humAb10.3. As this is the first description of monoclonal antibodies against the antigen MSP10, we cannot make this classification yet for the antibodies described here. The dissection of the actual mechanism would be a valuable subject of further investigations.

However, besides mere binding to the target, antibodies may also fulfil their function in vivo by initiating the complement cascade [63] and/or by recruiting immune effector cells. GIAs, such as the one used in this study, only address direct neutralization capacity. Therefore, it would make sense to perform tests which incorporate monocytes or neutrophil granulocytes [13, 64]. Additionally, it may be possible that the monoclonal antibodies characterized herein cannot completely inhibit the parasite invasion on their own. Rather, they could contribute to a synergistic neutralization when combined with antibodies of different anti-malarial specificities.

Besides a function during attachment to/invasion of new erythrocytes, the EGF-like domains of MSP10 might be carried into the erythrocyte during invasion and might have a function during the development of merozoites into rings. EGF-like domains of MSP1, MSP2 and MSP4 have been reported to stay attached to the merozoite membrane by GPI anchors after processing and to be carried into the freshly infected erythrocyte [11, 65, 66]. It has been assumed that some MSP1_19_-specific antibodies may compromise the intraerythrocytic development of Plasmodia [67, 68]. Thus, further studies may address the question if the C-terminal portion of MSP10, which comprises the EGF-like domains, also remains bound to the merozoite until after invasion. This might pave the way for the evaluation of MSP10 as a target of anti-malarial fusion proteins [69].

## Conclusion

The presented work describes the generation and characterization of three humAbs which are specific for the first or the second EGF-like domain of MSP10, respectively. To the best of our knowledge this study is the first to combine flow cytometric selection of antigen-specific B cells with EBV transformation and to successfully generate humAbs by this very technique. To our knowledge, these are the first human monoclonal antibodies directed at MSP10, which have been isolated. HumAb10.2 and humAb10.3 possess the genuine combination of corresponding heavy and light chains, respectively; they are highly specific for their corresponding plasmodial EGF-like domain and bind these with high affinity. In in vitro GIAs, the MSP10-specific humAbs demonstrated significant inhibition of the parasite strain 3D7A even though comparatively high antibody concentrations were required. Further experiments should address (1), the potential role of immune effector cells in inhibition of merozoites by MSP10-specific antibodies and (2), the identification of interaction partners of MSP10 to deepen understanding of its function(s). The generated humAbs represent a valuable tool to tackle these MSP10 related questions in the future.

## Abbreviations

Vh and Vl sequences: variable sequences of immunoglobulin heavy and light chains
EBV: Epstein-Barr virus
EGF: epidermal growth factor
LCL: lymphoblastoid cell line
humAb: human monoclonal antibody
CDR: complementarity determining region
EC_50_: half-maximal effective concentration
K_D_: dissociation constant
k_a_: association rate
k_d_: dissociation rate
IFA: immunofluorescence assay
GIA: growth inhibition assay
ELISA: enzyme-linked immunosorbent assay
GOI: gene of interest
